# Gabapentin and postoperative pain: a qualitative and quantitative systematic review, with focus on procedure

**DOI:** 10.1186/1471-2253-7-6

**Published:** 2007-07-07

**Authors:** Ole Mathiesen, Steen Møiniche, Jørgen B Dahl

**Affiliations:** 1 Department of Anaesthesia, Copenhagen University Hospital, Glostrup, Denmark

## Abstract

**Background:**

Gabapentin is an antiepileptic drug used in a variety of chronic pain conditions. Increasing numbers of randomized trials indicate that gabapentin is effective as a postoperative analgesic. This procedure-specific systematic review aims to analyse the 24-hour postoperative effect of gabapentin on acute pain in adults.

**Methods:**

Medline, The Cochrane Library and Google Scholar were searched for double-blind randomized placebo controlled trials of gabapentin for postoperative pain relief compared with placebo, in adults undergoing a surgical procedure.

Qualitative analysis of postoperative effectiveness was evaluated by assessment of significant difference (P < 0.05) in pain relief using consumption of supplemental analgesic and pain scores between study groups.

Quantitative analyses of combined data from similar procedures, were performed by calculating the weighted mean difference (WMD) of 24-hour cumulated opioid requirements, and the WMD for visual analogue scale (VAS) pain, (early (6 h) and late (24 h) postoperatively), between study groups. Side-effects (nausea, vomiting, dizziness and sedation) were extracted for calculation of their relative risk (RR).

**Results:**

Twenty-three trials with 1529 patients were included. In 12 of 16 studies with data on postoperative opioid requirement, the reported 24-hour opioid consumption was significantly reduced with gabapentin. Quantitative analysis of five trials in abdominal hysterectomy showed a significant reduction in morphine consumption (WMD – 13 mg, 95% confidence interval (CI) -19 to -8 mg), and in early pain scores at rest (WMD – 11 mm on the VAS, 95% CI -12 to -2 mm) and during activity (WMD -8 mm on the VAS; 95% CI -13 to -3 mm), favouring gabapentin. In spinal surgery, (4 trials), analyses demonstrated a significant reduction in morphine consumption (WMD of – 31 mg (95%CI – 53 to -10 mg) and pain scores, early (WMD – 17 mm on the VAS; 95 % CI -31 to -3 mm) and late (WMD -12 mm on the VAS; 95% CI -23 to -1 mm) also favouring gabapentin treatment. Nausea was improved with gabapentin in abdominal hysterectomy (RR 0.7; 95 % CI 0.5 to 0.9). Other side-effects were unaffected.

**Conclusion:**

Perioperative use of gabapentin has a significant 24-hour opioid sparing effect and improves pain score for both abdominal hysterectomy and spinal surgery. Nausea may be reduced in abdominal hysterectomy.

## Background

Prevention and treatment of postoperative pain and complications such as nausea and vomiting, continues to be a major challenge in postoperative care and plays an important role in the early mobilization and well-being of the surgical patient. Opioid analgesics, with their well-known side-effects, continues to represent a cornerstone in postoperative pain control, and testing new analgesics as well as combinations of analgesics in order to reduce the need for opioids, is a key area in acute pain research. [1]

Gabapentin, an anti-epileptic drug that has demonstrated analgesic effect in both diabetic neuropathy, post-herpetic neuralgia and neuropathic pain [2-4], affects the nociceptive process by binding to the α_2_δ subunit of voltage dependent calcium channels [5]. In pain models it has shown anti-hyperalgesic properties, possibly by reducing central sensitization, a prerequisite for postoperative hyperalgesia, and gabapentin, together with dextromethorphan and ketamine, represents a new option in postoperative pain care, which recently has been the subject of intensive research.

An increasing number of randomized trials indicate that gabapentin is effective as an postoperative analgesic. Until now, four meta-analyses with pooled data from rather few studies (7, 8, 12 and 16 trials, respectively) [6-9], demonstrates that gabapentin displays an effect on both postoperative pain score and opioid usage. In these meta-analyses, data from studies with very different surgical interventions are pooled and therefore the effect in a particular surgical setting is difficult to predict. We find that the recent number of publications allows a more procedure-specific systematic review in this area, which is the purpose of this paper.

## Methods

### Search strategy

Relevant randomized controlled trials were identified by performing a Medline [10], a Cochrane Library [11] and a Google Scholar search [12], without language restrictions. Free text combinations including the search terms: "gabapentin", "post-operative pain" and "post-operative analgesia" were used [see Additional file 1]. Additional papers where sought by reviewing the reference list of retrieved reports and relevant reviews. Last search was performed January 2007. The QOURUM guidelines for reporting meta-analyses were followed [13].

### Study selection criteria

Reports were considered if they were double-blind, randomized controlled trials of gabapentin (experimental intervention group) for postoperative pain relief compared with placebo (control intervention group) in adult patients (> 18 years) undergoing a surgical procedure. Only studies, in which data on either pain (visual analogue scale (VAS) or verbal score (VRS)) or supplemental postoperative analgesic consumption were stated, were included. Studies with less than 10 patients in treatment arms were not included [14].

### Assessment of quality

Each identified study was read and scored independently by two authors (OM + JBD), using a 5-point scoring system as described by Jadad et al [15]. If the reports were described as randomized, one point was given. One point was added if the randomization was described and appropriate (random number of tables, computer generated, etc), and likewise one point was subtracted if the randomization was described and inappropriate. If the study was described as double-blind one point was given, and an additional point was added if the method of blinding was described and appropriate. (identical placebo, active placebo etc.) For inappropriate blinding one point was subtracted. Finally a point was given if withdrawals and dropouts were appropriately described. Disagreement between the authors was solved through discussion.

### Extraction of data

Data from the studies were extracted onto a datasheet by one of the authors (OM). This included type of surgery; number of patients in intervention and control groups; time of administration and regimen of gabapentin treatment; mean VAS pain scores at rest and during mobilization early (at 4 or 6 hours) and late (at 24 hours) after surgery; supplemental analgesic regimen; type of and amount of supplemental analgesic consumption; and possible side-effects (nausea, vomiting, dizziness and sedation). Side-effects reported as somnolence or drowsiness were grouped under sedation, and reports of light-headedness and vertigo were grouped under dizziness. Pain reported on a 0 – 10 scale was converted to a 0 – 100 scale. In dose-finding studies, we extracted data from each dose-group onto the data sheet. When data in a study was only shown graphically, we extracted data from graphs. We contacted eight authors to get supplemental data for analysis, and received requested information from all.

### Qualitative analysis

Qualitative analysis of postoperative effectiveness was evaluated by assessment of significant difference (P < 0.05 as reported in the original paper) in pain relief using consumption of supplemental analgesic and pain scores between study groups, and by an assessment of clinical importance of observed findings. In addition, internal sensitivity was evaluated by an assessment of pain scores. It has been recognised that adequate sensitivity in trials of analgesics for acute pain, may only be achieved when patients are experiencing at least moderate pain (VAS pain score > 30 mm) with placebo, as it is difficult to detect an improvement with a low degree of pain [16,17].

### Quantitative analysis

Quantitative analyses of combined data were only performed with data from similar procedures (e.g. hysterectomy), but not across trials with different surgical procedures.

Quantitative analyses of combined data from similar procedures were performed by calculation of the weighted mean difference (WMD) of the 24-hour cumulated opioid use between study group, and by calculation of WMD of VAS pain scores between study groups early (4 – 6 h) and late (24 h) after surgery, whenever sufficient data were provided in original papers (e.g. standard deviation (SD), number of included patients in each study group and the relevant mean value).

Opioids other than morphine were converted to their morphine equivalents, based on the equivalence of 100 μg fentanyl, 5 mg ketobemidone, and 100 mg tramadol respectively to 10 mg morphine.

### Side-effects

From papers where data were available, dichotomous data were extracted for calculation of the relative risk (RR) of side-effects (nausea, vomiting, dizziness and sedation).

### Statistical software

Quantitative analyses were performed using the Review Manager (RevMan) software (version 4.2 for Windows, Copenhagen, The Nordic Cochrane Centre, The Cochrane Collaboration, 2003). A random effect model was used if the statistical test for heterogeneity was positive, and a fixed effect model if the test came out negative.

## Results

Our Medline and Cochrane Library search revealed 28 relevant randomized trials published in the period from 2001 to 2006. One additional paper was identified by the Google Scholar search, giving a total of 29 relevant trials. All trials were published in English.

Six trials were subsequently excluded. One [18] was only published in abstract form, one [19] had only 9 patients in the gabapentin treatment arm, one [20] addressed only chronic postoperative pain, and in two trials [21,22], gabapentin was part of a multimodal regimen in the treatment group, which was tested against placebo only. Finally, in one trial [23] oxazepam was used instead of placebo in the control group.

Thus, data from 23 trials including a total of 1529 patients, of which 810 received gabapentin were included.

One trial [24] tested four different dosing regimens of gabapentin versus placebo, one trial [25] tested two different dosing regimens versus placebo, and one [26] compared gabapentin administered before with after incision and placebo. Accordingly, 28 comparisons were performed in the 23 included trials.

### Characteristics of included trials

An overview of the included trials is presented in Table 1. The retrieved studies were generally of high quality (median quality score: 5; range 1 – 5).

**Table 1:** Included, randomized, double-blind, controlled studies of gabapentin in postoperative pain.

**Reference**	**Quality Score**	**Surgical procedure**	**η gabapentin/placebo**	**Gabapentin dosing & administration**	**Analgesic and delivery**	**Effect on analgesic consumption (24 hours)**	**Effect on pain score at rest (6 h)**	**Effect on pain score at rest (24 h)**	**Side-effects**
Dierking 2004 [27]	5	Abdominal hysterectomy	39/32	1200 mg 1 h pre-op. + 600 mg × 3	PCA – morphine	Morphine reduced from 63 to 43 mg	NS	NS	NS
Turan 2004a [28]	5	Abdominal hysterectomy	25/25	1200 mg 1 h pre-op.	PCA – tramadol intravenously	Tramadol reduced from 420 to 270 mg	VAS lower with gabapentin (P = 0.000)	VAS lower with gabapentin (P = 0.000)	NS
Gilron 2004 [30]	5	Abdominal hysterectomy	23/24	600 mg 1 h pre-op. + 600 mg × 2	PCA -morphine	Morphine reduced from 82 to 57 mg	VAS lower with gabapentin (P < 0.001)	VAS lower with gabapentin (P < 0.02)	Sedation increased with gabapentin
Turan 2006a [29]	5	Abdominal hysterectomy	25/25	1200 mg 1 h pre-op.	PCA -morphine	Morphine reduced from 53 to 41 mg	VAS lower with gabapentin (P < 0.01)	VAS lower with gabapentin (P < 0.05)	NS
Fassoulaki 2006 [31]	3	Abdominal hysterectomy	25/28	400 mg × 4 initiated day before surgery	PCA – morphine	Morphine reduced from 26 to 20 mg	NS	NS	
Pandey 2004a [32]	4	Lumbar discoidectomy	28/28	300 mg 2 h pre-op.	Fentanyl on demand	Fentanyl reduced from 360 to 234 ug	VAS lower with gabapentin (P < 0.05)	VAS lower with gabapentin (P < 0.05)	NS
Pandey 2005a [24]	5	Lumbar discoidectomy	4 × 20/20	300–600–900–1200 mg 2 h pre-op. (4 diff. groups)	PCA-fentanyl	Fentanyl reduced from 1218 to 627–988 ug	VAS lower with gabapentin (P < 0.05)	VAS lower with gabapentin (P < 0.05)	NS
Turan 2004b [33]	5	Lumbar discoidectomy or spinal fusion	25/25	1200 mg 1 h pre-op.	PCA-morphine	Morphine reduced from 43 to 16 mg	VAS lower with gabapentin. (P < 0.01)	NS	Vomiting reduced with gabapentin
Radhakrishnan 2005 [34]	4	Lumbar discoidectomy/laminectomy	30/30	400 mg night before surgery + 400 mg 2 h pre-op.	PCA-morphine (study lasted for 8 h)	NS (study lasted for 8 h)	NS		NS
Dirks 2002 [35]	5	Radical mastectomy	31/34	1200 mg 1 h preop.	PCA-morphine (study lasted for 4 h)	Morphine reduced from 29 to 15 mg	VAS lower with gabapentin (P < 0.018)		NS
Fassoulaki 2002 [36]	4	Matectomy or lumpectomy with axillary dissection	22/24	400 mg × 3 starting the evening before surgery	On demand. (Propoxyphene & paracetamol given i.m.)	NS	NS	NS	NS
Pandey 2005b [26]	5	Nefrectomy	2 × 20/20	Pre-incision (2 h pre-op.)/post-incision groups. 600 mg in both.	PCA-fentanyl	Fentanyl reduced from 925 to 563 ug/624 ug	VAS lower with gabapentin in both groups (P < 0.05)	VAS lower with gabapentin in both groups (P < 0.05)	NS
Bartholdy 2006 [38]	5	Laparascopic sterilization	38/38	1200 mg 1/2 h pre-op.	PCA-morphine (Study lasted for 4 h)	NS	NS	NS	NS
Pandey 2004b [37]	3	Laparascopic chole-cystectomy	153/153	300 mg 2 h pre-op.	Fentanyl on demand.	Fentanyl reduced from 356 to 221 ug	VAS lower with gabapentin (P < 0.05)	VAS lower with gabapentin (P < 0.05)	Sedation + PONV increased with gabapentin
Omran 2005 [39]	5	Pulmonal lobectomy	25/25	1200 mg 1 h pre-op. and 600 mg × 2	PCA-morphine	Morphine reduced from 32 to 24 mg	VAS lower with gabapentin (P < 0.05)	VAS lower with gabapentin (P < 0.05)	NS, vomiting reduced with gabapentin
Tuncer 2005 [25]	1	Major orthopedic surgery	2 × 15/15	1200 – 800 mg 1 h pre-op.	PCA-morphine (Study lasted only 4 hours)	Morphine reduced from 21 to 11 mg/15 mg	NS		NS
Menigaux 2005 [40]	5	Arthroscopic anterior cruciate ligament repair	20/20	1200 mg 1–2 h preop.	PCA-morphine	Morphine reduced from 48 to 21 mg	NS	NS	NS
Adam 2006 [41]	5	Arthropscopic shoulder surgery	27/26	800 mg 2 h pre-op.	Nerveblock + on demand paracetamol + propoxyphene	NS	NS	NS	NS
Turan 2007 [42]	5	Hand surgery	20/20	1200 mg 1 h pre-op.	IVRA + diclofenac according to VAS score	Diclofenac reduced from 63 to 30 mg	NS	NS	NS
Turan 2006b [43]	5	Lower extremity plastic surgery	20/20	1200 mg 1 h pre-op.	PCEA bolus (bupivacaine and fentanyl)	PCEA bolus reduced with gabapentin	VAS lower with gabapentin (P < 0.001)	NS	NS, dizziness increased with gabapentin
Al-Mujadi 2006 [45]	5	Thyroid surgery	37/35	1200 mg 2 h pre-op.	Morphine according to VAS score	Morphine reduced from 30 to 15 mg	VAS lower with gabapentin (P < 0.01)	VAS lower with gabapentin (P < 0.01)	NS
Mikkelsen 2006 [46]	5	Tonsillectomy	22/27	1200 mg 1 h pre-op. + 600 mg × 2	Morphine on demand + tbl. Ketobemidone by patient	Morphine NS. Ketobemidone reduced from 4.5 to 2.0 mg	NS	NS	Dizziness & vomiting increased with gabapentin
Turan 2004c [44]	5	Ear-Nose-Throat surgery	25/25	1200 mg 1 h pre-op.	Diclofenac according to VAS	Diclofenac reduced from 111 to 33 mg	VAS lower with gabapentin (P < = .001)	VAS lower with gabapentin (P < 0.001)	NS, dizziness increased with gabapentin

PCA, patient-controlled analgesia; NS, not significant; pre-op, pre-operatively; VAS, visual analogue scale; IVRA, intravenous regional anaesthesia; PCEA, patient-controlled epidural analgesia;

The surgical procedures were abdominal hysterectomy in five studies [27-31], spinal surgery in four studies [24,32-34], breast surgery in two studies [35,36] and a variety of different surgical procedures in the remaining twelve studies [25,26,37-46]. Median number of patients included in the studies was 50 (range 40 – 306). Three studies [25,35,38]lasted for only 4 h postoperatively, and one study [34] for 8 hours. The remaining studies lasted for 24 hours or longer.

### Gabapentin dosing

In most studies gabapentin was administered one to two hours preoperatively, but in three studies [31,34,36] a multiple (repeat) dosing regimen initiated the day before surgery was investigated.

In sixteen studies a single dose of gabapentin, varying from 300 mg to 1200 mg was administered and in seven studies [27,30,31,34,36,39,46] repeat dosing regimens were investigated. (Table 1)

### Postoperative analgesic effect: a qualitative overview

#### Supplemental opioid consumption

Supplemental analgesia was in fourteen trials administered as intravenous patient controlled analgesia (PCA), in four studies [32,36,37,46] on a demand basis, and in three studies [42,44,45] according to a VAS score. One study [43] used patient controlled epidural analgesia (PCEA) and finally in one study [41], the postoperative medication was administrated at home by the patient.

Morphine was used as postoperative analgesic in most studies, but four studies [24,26,32,37] used fentanyl, one study [28] tramadol intravenously, two studies [36,41] propoxiphen, two studies diclofenac [42,44] and one study [46] morphine for the first 4 hours succeeded by oral ketobemidon.

Sixteen studies provided data on 24-hour opioid consumption. The 24-hour morphine or calculated morphine equivalent usage was ranging from 4 to 99 mg in the treatment groups, and from 6 to 122 mg in the placebo groups, with a large variation between surgical procedures (Table 1).

**Figure 1:**
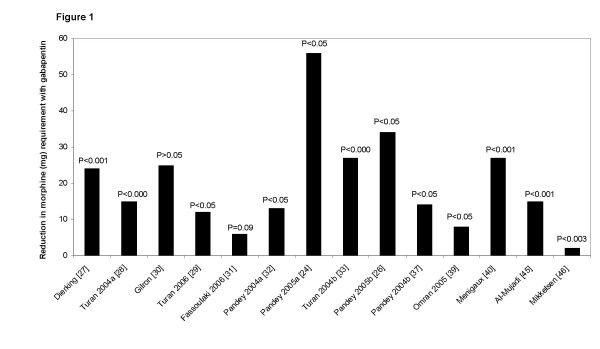
**Reduction in post-operative morphine requirements with gabapentin vs. placebo**. Data are calculated from the mean consumption of patient controlled analgesia (PCA) (21,23,24,25,26,27,28,30,36,37), 'on demand' administered analgesia (29,34) or analgesia 'administered at home by the patient' (43) in each study group from 0 to 24 h post-operatively.

In 12 of the 16 studies [24,26-29,32,33,37,39,40,45,46] opioid consumption was significantly reduced with gabapentin compared with placebo. Opioid sparing with gabapentin was, however, of variable clinical importance, varying between 2 and 59 mg of morphine (Figure 1). No obvious dose response relationship was apparent between significant and non-significant trials with respect to opioid sparing.

Two trials investigated gabapentin in a dose-response regimen. Pandey et al [24] found that increasing the gabapentin dose above 600 mg did not significantly increase the opioid sparing effect, and Tuncer et al [25] found no difference between 800 and 1200 mg gabapentin on morphine consumption.

### Pain intensity at rest

#### Early postoperatively

All 23 included trials reported on pain scores at rest early after operation. In twelve [24,26,28-30,32,33,37,39,43-45] of these 23 trials, significantly lower pain scores were observed with gabapentin. The reduction in pain ranged between 10 and 29 mm on the VAS score. None of the eleven trials with no observed reduction in pain intensity had sufficient internal sensitivity since pain scores in none of the control groups were more than 30 mm on the VAS.

#### Late postoperatively

Nineteen of the included 23 studies [24,26-33,36,37,39-46] reported on late (24 hours) pain score at rest. In ten of these trials [24,26,28-30,32,37,39,44,45] a significantly lower pain score, varying between a reduction of 5 and 23 mm on the VAS, was observed with gabapentin. Only one of the trials with no observed reduction in pain intensity had sufficient internal sensitivity.

No obvious dose response relationship was apparent between significant and non-significant trials with respect to late pain scores.

### Pain scores during activity

#### Early postoperatively

Eleven trials [27-29,31,34-36,38,39,45,46] reported on pain during activity early after operation. Five of these [28,29,35,39,45] observed a significant reduction with gabapentin, varying between 8 and 22 mm on the VAS. Four [27,31,34,36] of the six trials with no observed reduction in pain intensity had sufficient internal sensitivity.

#### Late postoperatively

Nine [27-31,36,39,45,46] trials reported on late pain score during activity and four [29,30,39,45] trials observed a significantly lower pain score with gabapentin, varying between 6 and 21 mm on the VAS score. Three [27,31,36] of the five trials with no observed reduction in pain intensity had sufficient internal sensitivity.

### Side-effects

#### Nausea

Twenty-one studies including 763 patients receiving gabapentin reported on postoperative nausea. In twenty of these no significant differences between study groups were observed, however, in one study on laparoscopic cholecystectomy [37] significantly more nausea and vomiting was observed in the gabapentin group.

#### Vomiting

Seventeen studies including 529 patients receiving gabapentin reported on vomiting and in 14 [24-29,32,34,38,40,42-45] of these no differences between groups were observed. One author found a higher [46] incidence and two authors a significant lower [33,39] incidence of vomiting with gabapentin.

#### Dizziness

Eighteen studies including 690 patients receiving gabapentin reported on this endpoint. Fourteen [24-29,32,33,35,38,39,41,42,45] found no difference in incidence between the groups. Three authors [43,44,46] found a higher incidence in the gabapentin group, and one [37] a higher incidence in the placebo group.

#### Sedation

Nineteen studies including 705 patients receiving gabapentin reported on the incidence of somnolence, drowsiness or sedation, and seventeen [24,26-29,33-35,38-46] found no difference between the two groups. Two authors [30,33] found a higher incidence of sedation with gabapentin.

In summary, a significant opioid-sparing effect was observed in almost all studies reporting of cumulative opioid consumption, which, however, was of varying magnitude. Pain scores at rest were, although to a smaller extent, significantly reduced with gabapentin in all studies but one, with sufficient sensitivity to test this parameter. Pain scores during activity were reduced in about half the sensitive studies. Finally, no consistent difference in opioid related side effects or other side effect attributable to gabapentin was observed in this qualitative analysis.

#### Procedure specific results

The 23 included studies represented a variety of surgical procedures of which more than one paper was available for only abdominal hysterectomy [27-31] spinal surgery (discectomy or spinal fusion surgery) [24,32-34] and mastectomy [35,36]. The remaining procedures were only evaluated in one study each.

In the following section we present quantitative analyses from studies of hysterectomy and spinal surgery, whereas data from studies of mastectomy did not allow for quantitative analyses.

#### Hysterectomy

Two studies administered a single dose of gabapentin preoperatively, and three studies used a multiple dosing regimen. (For details, see Table 1).

#### Opioid consumption

**Figure 2:**
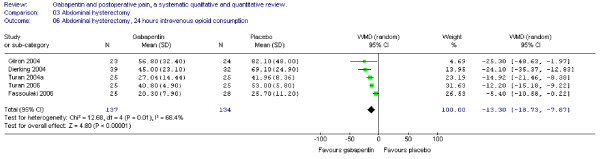
**Meta-analysis**. 24 hours cumulative morphine (mg) consumption for patients in abdominal hysterectomy receiving gabapentin preoperatively. WMD, weighted mean difference; CI, confidence interval.

Five trials of abdominal hysterectomy including 137 patients receiving gabapentin provided data on cumulative 24-hour supplemental opioid consumption. The combined data showed that the WMD of the 24-hour morphine consumption was significant in favour of gabapentin (WMD -13 mg, CI -19 to -8 mg). (Figure 2)

#### Pain scores

**Figure 3:**
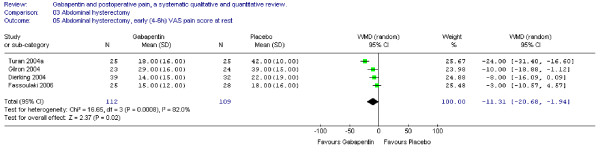
**Meta-analysis**. Visual analogue pain (VAS) score (0–100 mm) early (4–6 h) at rest for patients in abdominal hysterectomy receiving gabapentin preoperatively. WMD, weighted mean difference; CI, confidence interval.

Four trials including 112 patients receiving gabapentin provided data on early (4–6) VAS pain scores at rest. WMD between treatment groups was significant in favour of gabapentin (WMD -11 mm on the VAS; 95% CI -21 to -2 mm). (Figure 3)

**Figure 4:**
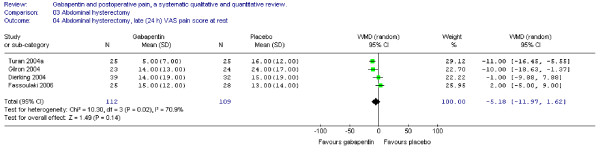
**Meta-analysis**. Visual analogue pain (VAS) score (0–100 mm) late (24 h) at rest for patients in abdominal hysterectomy receiving gabapentin preoperatively. WMD, weighted mean difference; CI, confidence interval.

The WMD of late (24 h) VAS pain scores between treatment groups was non-significant (WMD -5 mm on the VAS; 95% CI -12 to 2 mm). (Figure 4)

**Figure 5:**
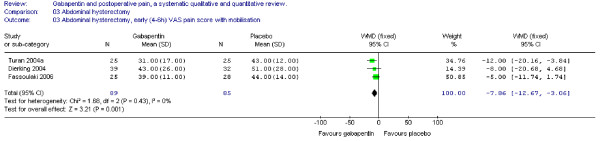
**Meta-analysis**. Visual analogue pain (VAS) score (0–100 mm) early (4–6 h) with mobilization for patients in abdominal hysterectomy receiving gabapentin preoperatively. WMD, weighted mean difference; CI, confidence interval.

**Figure 6:**
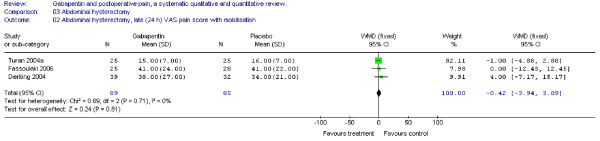
**Meta-analysis**. Visual analogue pain (VAS) score (0–100 mm) late (24 h) with mobilization for patients in abdominal hysterectomy receiving gabapentin preoperatively. WMD, weighted mean difference; CI, confidence interval.

Three trials provided data on early and late pain scores during activity respectively. The WMD of early VAS pain score between treatment groups was significant in favour of gabapentin (WMD -8 mm on the VAS; 95% CI -13 to -3 mm) (Figure 5), whereas the WMD of late VAS pain score between study groups was non-significant (WMD 0 mm on the VAS; 95% CI -4 to 3 mm) (Figure 6).

#### Nausea and vomiting

**Figure 7:**
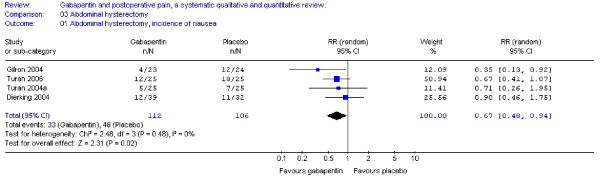
**Meta-analysis**. Side-effects, incidence of nausea for patients in abdominal hysterectomy receiving gabapentin preoperatively. RR, relative risk; CI, confidence interval.

**Figure 8:**
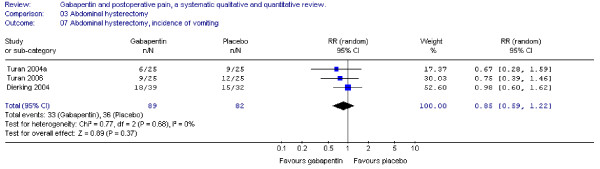
**Meta-analysis**. Side-effects, incidence of vomiting for patients in abdominal hysterectomy receiving gabapentin preoperatively. RR, relative risk; CI, confidence interval.

Four trials including 112 patients receiving gabapentin provided data on postoperative nausea, and the combined data showed that the RR was significant in favour of gabapentin (RR 0.7; 95 % CI 0.5 to 0.9) (Figure 7). Three trials provided data on postoperative vomiting. Calculation of the RR was non-significant (RR 0.9; 95% CI 0.6 to 1.2) (Figure 8).

#### Dizziness

**Figure 9:**
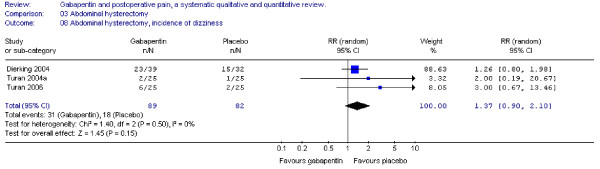
**Meta-analysis**. Side-effects, incidence of dizziness for patients in abdominal hysterectomy receiving gabapentin preoperatively. RR, relative risk; CI, confidence interval.

Three trials including 89 patients receiving gabapentin reported on the incidence of dizziness, and the combined data showed that the RR was non-significant (RR 1.4; 95% CI 0.9 to 2.1) (Figure 9).

#### Sedation

**Figure 10:**
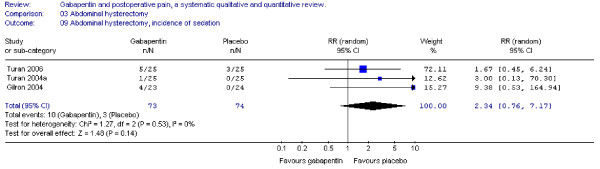
Side-effects, incidence of sedation for patients in abdominal hysterectomy receiving gabapentin preoperatively. RR, relative risk; CI, confidence interval.

Four trials investigated the incidence of either somnolence or sedation, and tree trials provided data for analyses. The combined data showed that the RR was non-significant between groups (RR 2.3; 95 % CI 0.8 to 7.2) (Figure 10).

In summary, cumulative 24-hour opioid consumption and early pain scores were significantly reduced with gabapentin. Nausea, but not vomiting, dizziness and sedation, was significantly reduced with gabapentin.

#### Spinal surgery

Three studies administered a single dose of preoperative gabapentin and one study a multiple doses regimen. (For details, see Table 1)

#### Opioid consumption

**Figure 11:**
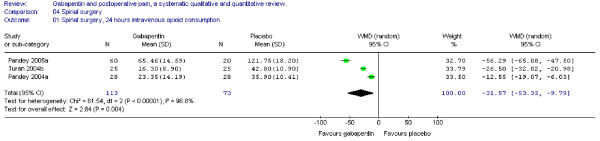
**Meta-analysis**. 24 hours cumulative morphine (mg) consumption for patients in spinal surgery receiving gabapentin preoperatively. WMD, weighted mean difference; CI, confidence interval.

Three trials of spinal surgery including 113 patients receiving gabapentin provided data on cumulative 24-hour supplemental opioid consumption. The combined data showed that the WMD between study groups was significant in favour of gabapentin (WMD -31 mg, 95% CI -53 to -10 mg) (Figure 11).

#### Pain scores

**Figure 12:**
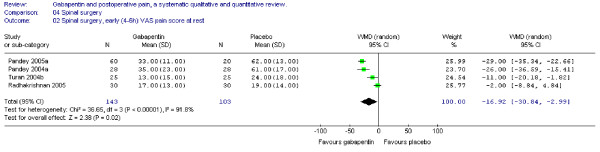
**Meta-analysis**. Visual analogue pain (VAS) score (0–100 mm) early (4–6 h) at rest for patients in spinal surgery receiving gabapentin preoperatively. WMD, weighted mean difference; CI, confidence interval.

Four trials including 143 patients receiving gabapentin provided data on early pain scores at rest, and the WMD was significant in favour of gabapentin (WMD -17 mm on the VAS; 95% CI -31 to -3 mm) (Figure 12).

**Figure 13:**
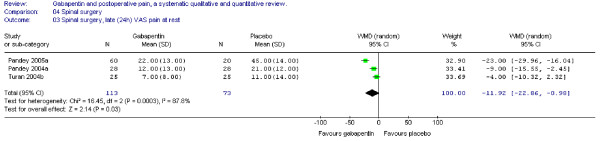
**Meta-analysis**. Visual analogue pain (VAS) score (0–100 mm) late (24 h) at rest for patients in spinal surgery receiving gabapentin preoperatively. WMD, weighted mean difference; CI, confidence interval.

In the same trials the WMD of late VAS pain scores at rest was also significant in favour of gabapentin (WMD -12 mm on the VAS; 95% CI -23 to -1 mm) (Figure 13).

Pain scores during activity early after operation were only provided in one trial [34] and found non-significant.

#### Nausea and vomiting

**Figure 14:**
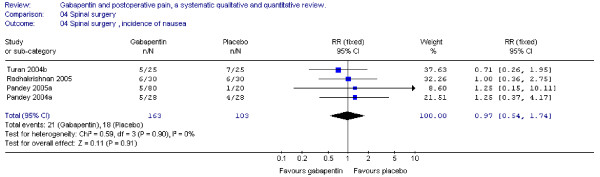
**Meta-analysis**. Side-effects, incidence of nausea for patients in spinal surgery receiving gabapentin preoperatively. RR, relative risk; CI, confidence interval.

Four trials provided data on postoperative nausea and vomiting. The combined data showed that the RR's were non-significant (nausea: RR 0.96; 95 % CI 0.47 to 1.97; vomiting: RR 0.51; 95 % CI 0.22 to 1.18) (Figure 14 + 15 ).

**Figure 15:**
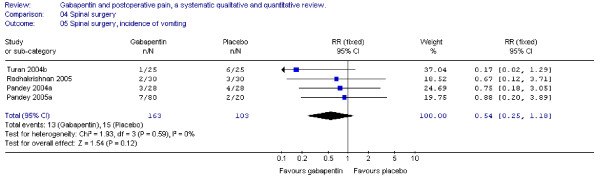
**Meta-analysis**. Side-effects, incidence of vomiting for patients in spinal surgery receiving gabapentin preoperatively. RR, relative risk; CI, confidence interval.

#### Dizziness

**Figure 16:**
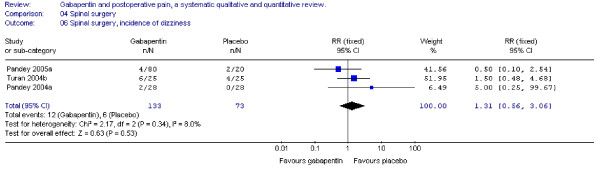
**Meta-analysis**. Side-effects, incidence of dizziness for patients in spinal surgery receiving gabapentin preoperatively. RR, relative risk; CI, confidence interval.

Three trials including 133 patients receiving gabapentin reported on the incidence of postoperative dizziness. Combined data showed that the RR was non-significant (RR 1.31; 95 % CI 0.6 to 3.1) (Figure 16).

#### Sedation

**Figure 17:**
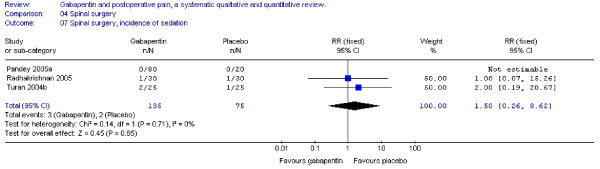
**Meta-analysis**. Side-effects, incidence of sedation for patients in spinal surgery receiving gabapentin preoperatively. RR, relative risk; CI, confidence interval.

Three trials including 135 patients receiving gabapentin provided data for the incidence of postoperative somnolence and sedation, and combined data showed that the RR was non-significant. (RR 1.5; 95 % CI 0.3 to 8.6). (Figure 17).

In summary, cumulative 24-hour opioid consumption, and early and late pain intensity at rest was significantly reduced with gabapentin. Nausea, vomiting dizziness and sedation was not affected by gabapentin.

## Discussion

This is the first systematic review with focus on procedure-specific effects of gabapentin in postoperative pain, and we have demonstrated that preoperative gabapentin reduces 24-hour postoperative opioid consumption for patients in abdominal hysterectomy and spinal surgery. For abdominal hysterectomy pain scores at rest and during activity were significantly improved with gabapentin in the early but not the late postoperative phase, whereas pain scores for patients in spinal surgery were improved at rest both early and late. Furthermore, an improvement in the incidence of nausea for abdominal hysterectomy patients was demonstrated, whereas no other side-effects (vomiting, dizziness, sedation) showed significant differences between treatment groups.

### Opioid consumption

The overall 24-hour reduction in opioid consumption following abdominal hysterectomy as well as spinal surgery, confirm the results from reviews with pooled data from a variety of surgical procedures [6-9], and the postoperative opioid sparing effect of gabapentin compared to placebo seems unquestionable.

In our review the opioid sparing in abdominal hysterectomy (13 mg morphine), is somewhat different from that of spinal surgery (32 mg morphine), and several reasons for this result could be thought of. First, data for spinal surgery might have been skewed, since one trial [24] used very high postoperative doses of fentanyl, and consequently reported a larger opioid sparing effect (56 mg morphine). If this study was removed from the analyses, we could demonstrate a 24-hour WMD of – 20 mg morphine instead. Second, the pain scores at rest for patients in spinal surgery are higher than for abdominal hysterectomy both early (mean VAS 42 mm vs. 30 mm) and late (mean VAS 26 mm vs. 17 mm), and it is possible that the analgesic effect of gabapentin simply reflects this difference in pain states. Third, the patho-physiology of postoperative pain in spinal surgery might be of a different origin than for abdominal hysterectomy, e.g. that pre- and per-operative nerve damage in a higher degree contributes to postoperative pain in spinal surgery than in abdominal hysterectomy. Gabapentin is effective in the treatment of chronic neuropathic pain [4], and so a larger analgesic effect in postoperative pain after spinal surgery than abdominal hysterectomy is a possibility.

### Pain score

The relevance of the pain score improvement demonstrated in the quantitative analysis is debatable. A minimal clinical significant change in patient pain severity has previously been measured as 13 mm in trauma patients [47,48], and another author [49] reports of a 33 % pain intensity difference being clinically meaningful. Using these definitions our 11 mm VAS pain relief at rest for abdominal hysterectomy patients are of minor clinical value, whereas the pain relief in spinal surgery patients (- 17 mm at rest early) could be of clinical importance, since it may improve patients ability to resume daily activity.

However, comparison of pain scores during PCA treatment is problematic since the patients can avoid uncontrolled pain in both treatment groups. Accordingly, our primary outcome parameter was morphine consumption.

Our analyses indicate, that preoperative gabapentin reduces postoperative pain scores more effectively in the early rather than the late postoperative period. Several explanations may be possible. Most studies used a single dose of gabapentin, and since T_max _in plasma is 2–3 h, the plasma level of gabapentin accordingly is higher in the early postoperative phase. One study [27] has demonstrated a significant inverse association between plasma levels of gabapentin and morphine usage both at two and four hours postoperatively, indicating a dose-response relationship. Furthermore, the postoperative pain scores are generally higher in the early compared to the late postoperative phase, and consequently, the improvement in pain score by gabapentin could also be relatively larger in this phase. Finally, the absence of internal sensitivity (VAS < 30 mm) may also play a role in measuring the effect of gabapentin, since five of eight trials in the two procedures that were non-significant had low internal sensitivity.

In the most recent review [9] the authors reported of a significant reduction in both early (6 h) and late (24 h) VAS pain scores with a single preoperative dose of 1200 mg gabapentin (WMD -17 mm and -11 mm, respectively). The improvement in VAS-pain reported in the reviews by Hurley et al [8] and Seib et al [7] also correlate well with our findings.

### Side-effects

One of the major challenges in postoperative pain treatment is to combine different treatment modalities, in order to improve patient analgesia and potentially reduce side-effects [1]. A cornerstone in this area is to minimize the need for postoperative opioid analgesics and hopefully reduce side effects, especially postoperative nausea and vomiting (PONV). Zhao et al has reported a dose-response relationship between opioid use and adverse events for patients in ambulatory surgery. Every 3–4 mg increase of morphine usage was associated with 1 additional clinically meaningful opioid-related symptom [50]. Marret et al [51] reported that the opioid sparing by NSAID and COX-2 inhibitors (approximately 30 %) was followed by a significant reduction in PONV and sedation (also by approximately 30%), but not in urinary retention and respiratory depression.

In this light it is noteworthy, that despite of the opioid sparing effect with gabapentin, our analyses of side-effects showed a just significant lower incidence of nausea in favour of gabapentin for abdominal hysterectomy, but not spinal surgery patients, while vomiting and sedation were non-significant between treatment groups. These disappointing results correspond with results from another review on opioid sparing with the use of COX-2 inhibitors and the lack of evidence for reduction of opioid related side-effects [52].

One of the reasons for this finding could be the relatively small number of patients and thereby low statistical power of our analyses. A post-hoc statistical power calculation of the non-significant quantitative analyses of adverse events, using a risk of Type I error on 5% and the observed cumulated difference between treatment groups, revealed a statistical power of less than 50% on analyses of most adverse events in both abdominal and spinal surgery.

Another reason could be the lack of quality of data on adverse events from the original papers, as in most reports adverse events were not well defined and only reported as present or absent. Furthermore, data of adverse events were a mixture of spontaneous reporting and specific questioning.

We did not find any sign of clinically limiting side-effect with the use of gabapentin, and especially the data for sedation and dizziness were non-significant. It is possible that the well-known sedative effect of gabapentin may be masked by the use of opioids in the studies and the fact that we monitor patients in the early phase after general anaesthesia.

### Limitations

The wide variability of gabapentin dosing regimens, and the differences in pain score- and side-effect evaluation, undoubtedly influences the outcome of this review, as do the pooling of different rescue analgesics (morphine, fentanyl and tramadol). By making this review procedure specific we tried to minimize variability in the pooled outcome parameters, but differences in e.g. surgical techniques among the different centres may still limit our analyses.

Another important limitation for the interpretation of the analgesic effect of gabapentin is the low VAS pain score in many of the included trials, both early and especially 24 hour postoperatively, since previous studies has emphasized the importance of moderate to high initial pain intensity in postoperative assessment of analgesic drugs [16].

In our quantitative analysis the primary outcome is PCA or "on demand" opioid consumption with simultaneous VAS-pain measurement. The assumption is that patients in the active and control treatment groups will titrate "down" to the same low VAS-pain, and the difference in opioid consumption at the same VAS-pain gives us the valid difference in opioid consumption. This only happens in three of nine trials which is a limitation when the analgesic effect of gabapentin is interpreted.

All trials in this review were randomized and the methodological quality satisfactory, the median Oxford quality score being 5, and so selection bias should not have been a problem. Most trials reported of a positive outcome of either opioid reduction or pain score improvement, and publication bias (skewed publication of only trials with a positive outcome) cannot be ruled out. Given the nature of abdominal hysterectomy gender bias is not the case here, whereas for the spinal surgery trials twice as many male patients were included as women (173 vs. 93). However, since the male/female distribution generally was equal among treatment groups, we find no tendency to gender bias in the trials. Language restriction to only articles published in English could be a potential bias, but we did not find any non-English published articles. Many analyses revealed heterogeneity among the included studies, and in these cases we used a random-effect model in the analyses to compensate. Finally almost all studies represent small sample sizes with the increased likelihood of Type 2 errors.

The development of chronic post surgical pain has attracted increased attention [53] and with central sensitization as a prerequisite, gabapentin with its ability to attenuate secondary hyperalgesia in pain models [54,55], is an interesting "anti-hyperalgesic". Only one of the included trials investigated pain beyond the immediate postoperative period [31], and our review cannot make a conclusion on this subject. In two excluded studies by Fassoulaki et al [21,22], gabapentin was investigated together with local anaesthetics as part of a multimodal analgesic regimen. In both abdominal hysterectomy and breast surgery, a reduction in postoperative morphine usage, as well as pain for more than one month postoperatively was reported.

In the qualitative analysis of the trials we found no obvious dose-response relationship for the use of perioperative gabapentin. One trial [24] investigated the effect of different preoperative gabapentin doses (300–600–900–1200 mg) and found no additional analgesic effect raising the gabapentin dose above 600 mg. It is the only study in this review addressing this subject and so, the material does not allow for any conclusions on this topic.

In a recent pilot trial by Leung and colleagues [19], preoperative gabapentin significantly reduced the incidence of postoperative delirium. This subject is sparsely addressed in the trials covered here. Two studies reported on lack of concentration [24,26], two studies reported on visual disturbances [35,39], and one study on hallucinations [43]. All were non-significant on the subject.

## Conclusion

In conclusion, the perioperative use of gabapentin has a significant 24-hour opioid sparing effect for both abdominal hysterectomy and spinal surgery patients, whereas the reduction in pain score is more inconsistent. Nausea may be reduced in abdominal hysterectomy. All other side-effects were non-significant between treatment groups.

Future trials in this area should include gabapentin as part of a multimodal postoperative treatment strategy, with focus on both acute and chronic pain states. Pain score during mobilisation is mandatory, and the effect of gabapentin on postoperative delirium needs further exploration.

## Competing interests

Dr. Dahl has received an unrestricted research grant from Pfizer, Denmark.

## Authors' contributions

OM was involved with searching, data extraction, quality scoring, analysis and writing, SM was involved with analysis, writing and JBD was involved with quality scoring and writing.

## Pre-publication history

The pre-publication history for this paper can be accessed here:



## Supplementary Material

Additional file 1 Search strategy. the search strategy for trials included in the review. Click here for file

## References

[B1] Kehlet H, Dahl JB (2003). Anaesthesia, surgery, and challenges in postoperative recovery. The Lancet.

[B2] Backonja M, Beydoun A, Edwards KR, Schwarts SL, Fonseca V, Hes M, LaMoreaux L, Garofalo E (1998). Gabapentin for the symptomatic treatment of painful neuropathy in patients with diabetes mellitus: a randomised controlled trial. JAMA.

[B3] Rowbotham M, Harden N, Stacey B, Bernstein P, Magnus Muller L (1998). Gabapentin for the treatment of postherpetic neuralgia. JAMA.

[B4] Serpell MG (2002). Gabapentin in neuropathic pain syndromes: a randomised, double-blind, placebo-controlled trial. Pain.

[B5] Maneuf YP, Gonzalez MI, Sutton KS, Chung FZ, Pinnock RD, Lee K (2003). Cellular and molecular action of the putative GABA-mimetic, gabapentin. Cell Mol Life Sci.

[B6] Dahl JB, Mathiesen O, Møiniche S (2004). "Protective premedication": an option with gabapentin and related drugs? A review of gabapentin and pregabalin in the treatment of post-operative pain. Acta Anaesthesiol Scand.

[B7] Seib RK, Paul JE (2006). Preoperative gabapentin for postoperative analgesia: a meta-analysis. Can J Anesth.

[B8] Hurley RW, Cohen SP, Williams KA, Rowlingson AJ, Wu CL (2006). The analgesic effects of perioperative gabapentin on postoperative pain: a meta-analysis. Reg Anesth Pain Med.

[B9] Ho K-Y, Gan TJH, Habib AS (2006). Gabapentin and postoperative pain – a systematic review of randomised controlled trials. Pain.

[B10] Medline. http://www.ncbi.nlm.nih.gov.

[B11] Cochrane Library. http://www.thecochranelibrary.com.

[B12] Google Scholar. http://www.scholar.google.com.

[B13] Moher D, Cook DJ, Eastwood S, Olkin I, Rennie D, Stroup DF, for the QOURUM Group (1999). Improving the quality of reports of meta-analyses of randomised controlled trials: the QUOROM statement. Lancet.

[B14] L'Abbé KA, Detsky AS, O'Rourke K (1987). Meta-analysis in clinical research. Ann Intern Med.

[B15] Jadad AR, Moore A, Carroll D, Jenkinson C, Reynolds DJM, Gavaghan DJ, McQuay HJ (1996). Assessing the quality of reports of randomized clinical trials: is blinding necessary?. Control Clin Trials.

[B16] Bjune K, Stubhaug A, Dodgson MS, Breivik H (1996). Additive analgesic effect of codeine and paracetamol can be detected in strong, but not moderate, pain after Caesarean section. Baseline pain-intensity is a determinant of assay-sensitivity in a postoperative analgesic trial. Acta Anesthesiol Scand.

[B17] Collins SL, Moore RA, McQuay HJ (1997). The visual analogue pain intensity scale: what is moderate pain in millimetres?. Pain.

[B18] Gregg AK, Francis S, Sharpe P, Rowbotham DJ (2001). Analgesic effect of gabapentin premedication in laparoscopic cholecystectomy: a randomised double-blind placebo-controlled trial [abstract]. Br J Anaesth.

[B19] Leung JM, Sands LP, Rico M, Petersen KL, Rowbotham MC, Dahl JB, Ames C, Chou D, Weinstein P (2006). Pilot clinical trial of gabapentin to decrease postoperative delirium in older patients. Neurology.

[B20] Sihoue ADL, Lee TW, Wan IYP, Thung KH, Yim APC (2006). The use of gabapentin for post-traumatic pain in thoracic surgery patients. Eur J Cardiothorac Surg.

[B21] Fassoulaki A, Triga A, Melemeni A, Sarantopoulos C (2005). Multimodal analgesia with gabapentin and local anesthetics prevents acute and chronic pain after breast surgery for cancer. Anesth Analg.

[B22] Fassoulaki A, Melemeni A, Stamatakis E, Petropoulos G, Sarantopoulos C (2007). A combination of gabapentin and local anaesthetics attenuates acute and late pain after abdominal hysterectomy. Eur J Anaesth.

[B23] Rorarius MGF, Mennander S, Suominen P, Rintala S, Puura A, Pirhonen R, Salmelin R, Haanpää M, Kujansuu E, Yli-Hankala A (2004). Gabapentin for the prevention of postoperative pain after vaginal hysterectomy. Pain.

[B24] Pandey CK, Navkar DV, Giri PJ, Raza M, Behari S, Singh RB, Singh U, Singh PK (2005). Evaluation of the optimal preemptive dose of gabapentin for postoperative pain relief after lumbar diskectomy. J Neurosurg Anesthesiol.

[B25] Tuncer S, Bariskaner H, Reisli R, Sarkilar G, Cicekci F, Otelcioglu S (2005). Effect of gabapentin on postoperative pain: A randomized, placebo-controlled clinical study. The pain clinic.

[B26] Pandey CK, Singhal V, Kumar M, Lakra A, Ranjan R, Pal R, Raza M, Singh U, Singh PK (2005). Gabapentin provides effective postoperative analgesia whether administered pre-emptively or post-incision. Can J Anesth.

[B27] Dierking G, Duedahl TH, Rasmussen ML, Fomsgaard JS, Møiniche S, Rømsing J, Dahl JB (2004). Effects of gabapentin on postoperative morphine consumption and pain after abdominal hysterectomy: A randomised, double-blind trial. Acta Anaesthesiol Scand.

[B28] Turan A, Karamanlioğlu B, Memiş D, Usar P, Pamukcu Z, Türe M (2004). The analgesic effects of gabapentin after total abdominal hysterectomy. Anesth Analg.

[B29] Turan A, White PF, Karamanlioğlu B, Memis D, Taşdoğan M, Pamukcu Z, Yavuz E (2006). Gabapentin: an alternative to the cyclooxygenase-2 inhibitors for perioperative pain management. Anesth Analg.

[B30] Gilron I, Orr E, Tu D, O'Neill P, Zamora JE, Bell AC (2005). A placebo-controlled randomized clinical trial of perioperative administration of gabapentin, rofecoxib and their combination for spontaneous and movement-evoked pain after abdominal hysterectomy. Pain.

[B31] Fassoulaki A, Stamatakis E, Petropoulos G, Siafaka I, Hassiakos D, Sarantopoulos C (2006). Gabapentin attenuates late but not acute pain after abdominal hysterectomy. Euro J Anaesth.

[B32] Pandey CK, Sahay S, Gupta D, Ambesh SP, Singh RB, Raza M, Singh U, Singh PK (2004). Preemptive gabapentin decreases postoperative pain after lumbar discoidectomy. Can J Anesth.

[B33] Turan A, Karamanlioğlu B, Memiş D, Hamamcioglu MK, Tükenmez B, Pamukcu Z, Kurt I (2004). Analgesic effects of gabapentin after spinal surgery. Anesthesiology.

[B34] Radhakrishnan M, Bithal PK, Chaturvedi A (2005). Effect of preemptive gabapentin on postoperative pain relief and morphine consumption following lumbar laminectomy and discectomy. J Neurosurg Anesthesiol.

[B35] Dirks J, Fredensborg BB, Christensen D, Fomsgaard JS, Flyger H, Dahl JB (2002). A randomized study of the effects of single-dose gabapentin versus placebo on postoperative pain and morphine consumption after mastectomy. Anesthesiology.

[B36] Fassoulaki A, Patris K, Sarantopoulos C, Hogan Q (2002). The analgesic effect of gabapentin and mexiletine after breast surgery for cancer. Anesth Analg.

[B37] Pandey CK, Priye S, Singh S, Singh U, Singh RB, Singh PK (2004). Preemptive use of gabapentin significantly decreases postoperative pain and rescue analgesic requirements in laparoscopic cholecystectomy. Can J Anesth.

[B38] Bartholdy J, Hilsted KL, Hjortsø NC, Engbæk J, Dahl JB (2006). Effect of gabapentin on morphine demand and pain after laparoscopic sterilization using Filshie clips. A double blind randomized clinical trial. BMC Anesthesiol.

[B39] Omran AF, Mohamed AE (2005). A randomized study of the effects of gabapentin versus placebo on post-thoracotomy pain and pulmonary function. Eg J Anaesth.

[B40] Menigaux C, Adam F, Guignard B, Sessler DI, Chauvin M (2005). Preoperative gabapentin decreases anxiety and improves early functional recovery from knee surgery. Anesth Analg.

[B41] Adam F, Menigaux C, Sessler DI, Chauvin M (2006). A single preoperative dose of gabapentin (800 milligrams) does not augment postoperative analgesia in patients given interscalene brachial plexus blocks for arthroscopic shoulder surgery. Anesth Analg.

[B42] Turan A, White PF, Karamanlioğlu B, Pamukcu Z (2007). Premedication with gabapentin: the effect on tourniquet pain and quality of intravenous regional anaesthesia. Anesth Analg.

[B43] Turan A, Kaya G, Karamanlioğlu B, Pamukcu Z, Apfel CC (2006). Effect of oral gabapentin on postoperative epidural analgesia. Br J Anaesth.

[B44] Turan A, Memiş D, Karamanlioğlu B, Yağiz R, Pamukcu Z, Yavuz E (2004). The analgesic effects of gabapentin in monitored anesthesia care for ear-nose-throat surgery. Anesth Analg.

[B45] Al-Mujadi H, A-Refai AR, Katzarov MG, Dehrab NA, Batra YK, Al-Qattan AR (2006). Preemptive gabapentin reduces postoperative pain and opioid demand following thyroid surgery. Can J Anesth.

[B46] Mikkelsen S, Hilsted KL, Andersen PJ, Hjortsø NC, Enggaard TP, Jørgensen DG, Hansen M, Henriksen J, Dahl JB (2006). The effect of gabapentin on post-operative pain following tonsillectomy in adults. Acta Anaesthesiol Scand.

[B47] Todd KH, Funk KG, Funk JP, Bonacci R (1996). Clinical significance of reported changes in pain severity. Ann Emerg Med.

[B48] Gallagher EJ, Liebman M, Bijur PE (2001). Prospective validation of clinically important changes in pain severity measured on a visual analog scale. Ann Emerg Med.

[B49] Farrar JT, Portenoy RK, Berling JA, Kinman JL, Strom BL (2000). Defining the clinically important difference in pain outcome measures. Pain.

[B50] Zhao SZ, Chung F, Hanna DB, Raymundo AL, Cheung RY, Chen C (2004). Dose-response relationship between opioid use and adverse effects after ambulatory surgery. J Pain Symptom Manage.

[B51] Marret E, Kurdi O, Zufferey p, Bonnet F (2005). Effects of nonsteroidal antiinflammatory drugs on patient-controlled analgesia morphine side effects. Anesthesiology.

[B52] Romsing J, Møiniche S, Mathiesen O, Dahl JB (2005). Reduction of opioid-related adverse events using opioid-sparing analgesia with COX-2 inhibitors lacks documentation: a systematic review. Acta Anaesthesiol Scand.

[B53] Kehlet H, Jensen TS, Woolf CJ (2006). Persistent postsurgical pain: risk factors and prevention. Lancet.

[B54] Mathiesen O, Imbimbo BP, Hilsted KL, Fabbri L, Dahl JB (2006). CHF3381, a N-methyl-D-aspartate receptor antagonist and monoamine oxidase-A inhibitor, attenuates secondary hyperalgesia in a human pain model. J Pain.

[B55] Dirks J, Petersen KL, Rowbotham MC, Dahl JB (2002). Gabapentin suppresses cutaneus hyperalgesia following heat-capsaicin sensitization. Anesthesiology.

