# Supplementary material for: Gabapentin and postoperative pain: a qualitative and quantitative systematic review, with focus on procedure
Source: BMC Anesthesiol. 2007 Jul 7;7:6. doi: 10.1186/1471-2253-7-6 (PMC1950698; doi:10.1186/1471-2253-7-6)
Supplement: Additional file 1 — Search strategy. the search strategy for trials included in the review. [file 1471-2253-7-6-S1.doc]

The following search strategy was used in Medline, and modified in the other databases to find the relevant trials.

#1 Gabapentin

#2 Randomised controlled trials

#3 Postoperative pain

#4 Pain OR analgesia

#5 #3 OR #4

#6 #1 AND #2 AND #5
